# Host’s genetic background determines the outcome of reciprocal faecal transplantation on life-history traits and microbiome composition

**DOI:** 10.1186/s42523-022-00210-y

**Published:** 2022-12-23

**Authors:** Heli Juottonen, Neda N. Moghadam, Liam Murphy, Johanna Mappes, Juan A. Galarza

**Affiliations:** 1 Department of Biological and Environmental Sciences, University of Jyväskylä, P.O. Box 35, 40014 Jyväskylä, Finland; 2 Organismal and Evolutionary Biology Research Program, Faculty of Biological and Environmental Sciences, University of Helsinki, Viikki Biocenter 3, 00014 Helsinki, Finland

**Keywords:** Lepidoptera, *Arctia plantaginis*, Wood tiger moth, Bacterial community, Growth, Genotype, 16S rRNA, Gut, Long amplicon

## Abstract

**Background:**

Microbes play a role in their host's fundamental ecological, chemical, and physiological processes. Host life-history traits from defence to growth are therefore determined not only by the abiotic environment and genotype but also by microbiota composition. However, the relative importance and interactive effects of these factors may vary between organisms. Such connections remain particularly elusive in Lepidoptera, which have been argued to lack a permanent microbiome and have microbiota primarily determined by their diet and environment. We tested the microbiome specificity and its influence on life-history traits of two colour genotypes of the wood tiger moth (*Arctia plantaginis*) that differ in several traits, including growth. All individuals were grown in the laboratory for several generations with standardized conditions. We analyzed the bacterial community of the genotypes before and after a reciprocal frass (i.e., larval faeces) transplantation and followed growth rate, pupal mass, and the production of defensive secretion.

**Results:**

After transplantation, the fast-growing genotype grew significantly slower compared to the controls, but the slow-growing genotype did not change its growth rate. The frass transplant also increased the volume of defensive secretions in the fast-growing genotype but did not affect pupal mass. Overall, the fast-growing genotype appeared more susceptible to the transplantation than the slow-growing genotype. Microbiome differences between the genotypes strongly suggest genotype-based selective filtering of bacteria from the diet and environment. A novel cluster of insect-associated *Erysipelotrichaceae* was exclusive to the fast-growing genotype, and specific *Enterococcaceae* were characteristic to the slow-growing genotype. These *Enterococcaceae* became more prevalent in the fast-growing genotype after the transplant, which suggests that a slower growth rate is potentially related to their presence.

**Conclusions:**

We show that reciprocal frass transplantation can reverse some genotype-specific life-history traits in a lepidopteran host. The results indicate that genotype-specific selective filtering can fine-tune the bacterial community at specific life stages and tissues like the larval frass, even against a background of a highly variable community with stochastic assembly. Altogether, our findings suggest that the host's genotype can influence its susceptibility to being colonized by microbiota, impacting key life-history traits.

**Supplementary Information:**

The online version contains supplementary material available at 10.1186/s42523-022-00210-y.

## Background

Variation in traits within a population can be partly determined by genetic polymorphisms. Uncovering genotype–phenotype associations allows the analysis of the evolution and adaptive advantages of the traits. It is increasingly recognized that phenotype may also be influenced by the microbiome, which all animals, including insects, possess [20]. In general, the microbiome can potentially influence the host’s life history and fitness [31, 58, 99]. In insects, the microbiome has been related to behavioural, nutritional, and life-history traits [11, 114, 119]. Moreover, microbiome composition can vary according to the host’s genetic background [58, 119]. For instance, the gut microbiome can mediate genotype effects on the phenotype: In *Drosophila*, the host genotype influences the microbiome composition, leading to differences in nutrition between phenotypes [11].

A stable, symbiotic microbiome can confer benefits on the insect host, such as aiding its growth [18, 41, 48]. On the other hand, even mutualistic symbionts incur costs [75], and opportunistic pathogenic bacteria can severely disadvantage the host [39, 97]. The outcome of these associations can depend on genotype-genotype interactions between the microbe and its host [79], as well as among microbes [55, 98]. For example, in the pea aphid (*Acyrthosiphon pisum*), the host’s genotype influences the protection given by bacterial symbionts against pathogens [79, 121]. In turn, host-to-microbe effects can play an important role in microbiome assembly in the host [23]. Selective mechanisms that impact the establishment of microbes in insects include specialized organs [53, 73] and mechanisms that vary with host genetic background, such as innate immunity [54, 72]. Such filtering due to host traits and genetic background could influence the host’s fitness and life-histories [52].

The gut bacteria of lepidopteran larvae show metabolic potential to benefit the host by digesting and detoxifying food plants [117, 124] and by producing antimicrobial compounds against invaders [92]. However, disruptions of the gut during moulting and metamorphosis, a highly alkaline pH (up to 11–12), lack of specialized gut structures, and fast passage of food can constrain the development of a consistent symbiotic microbiome [20]. Accordingly, several studies have concluded that there is no stable microbiome in Lepidoptera [35, 61, 103]. Despite reports on the effects of diet, habitat, and developmental stage on gut bacteria [7, 29, 44, 92, 93, 103], no clear consensus exists on the ecological roles of bacteria in Lepidoptera [77].

Lepidopteran larval growth has been found to be correlated [90] and not correlated [12] with microbiome composition. Antibiotic treatment of lepidopteran larvae has similarly led to increased growth [27, 116], decreased growth [124], or no effect on growth [35]. Moreover, increased growth has been observed in axenic larvae [62]. Thus, the causal connections between microbiota and Lepidoptera growth traits remain elusive. One way to identify such connections is through microbiota transplants. Transplants (or bacteriotherapy) have been extensively applied in the biomedical field to study the potential of microbes to impact health and disease [1, 118, 126]. The principle is to transfer microbes from a healthy individual to an unhealthy individual aiming to enrich beneficial microbes and restore a balanced microbiome. In insects, gut microbiota transplants are starting to reveal the importance of microbes in host development, immune response, and survival in dung beetles, cockroaches, bumblebees and parasitoid wasps [42, 68, 78, 80, 113]. However, such studies are lacking in Lepidoptera, one of the most species-rich and ecologically important groups of insects, in which less than 0.1% of species have been screened for microbes [77]. The uncertainties of the functional role and specificity of microbes in Lepidoptera make this group a particularly important target for transplant experiments.

Here, we investigate the effect of microbiome transplantation on wood tiger moths (*Arctia plantaginis*) with distinct genetic backgrounds and life-history traits. A reciprocal faecal transplantation was carried out between wood tiger moths of two colour genotypes that differ in the duration of their larval stage by adding frass (i.e., larval faeces) to their diet. The experimental insects have been reared in the laboratory for several generations, kept in similar conditions and fed the same diet. We followed the microbiome’s compositional changes during larval development and in the resulting adults. Thus, any consistent microbiome differences between host genotypes could reflect selective filtering of bacteria. We ask (i) if each genotype has its own associated microbiome, (ii) if it is stable across life-stages, and (iii) if microbiome transplant can reverse the growth rate between the genotypes. We also examine if the transplantation impacts other important fitness traits, such as pupal mass and the volume of defensive secretions.

## Methods

### Study species and genotype lines

The wood tiger moth is an aposematic species distributed throughout the Holarctic [37]. In Europe, males display colour polymorphism, having yellow or white hindwings and co-occur at variable frequencies within populations [25, 37]. The two colour morphs differ in key fitness traits such as mating success [30, 69], immune responses [70], protection against predators [56, 87], and flight activity [88]. The yellow-white hindwing polymorphism is determined by a single Mendelian locus with two alleles in which the yellow allele (y) is recessive to the white (W) allele [71]. Hence, the white colouration is produced by WW and Wy allelic combinations, whereas yy produces yellow. Analyses of selection lines show that the homozygous genotypes differ in the length of their larval stage (i.e., from egg hatching to pupation). Individuals of the WW genotype have a significantly shorter larval stage than those of the yy genotype (Additional file 1: Fig. S1). Adults of both colour morphs release defensive secretions from their anal cavity, which are effective at deterring invertebrate predators, with yellows having stronger chemical defence than whites [86]. Thus, this species offers a good opportunity to study the impact of microbes on life-history and fitness traits in relation to the host’s genetic background.

### Larval sampling and rearing before frass transplant

Genotype selection lines of wood tiger moths have been maintained for over 12 generations at the University of Jyväskylä, Central Finland. For this study, we selected four families of WW and four of yy genotypes to characterize their bacterial communities as they develop with or without faecal transplantation (see below). We included four families to cover for possible variation among families in the analysis of genotype effects, and we did not analyze family effects. The general rearing protocol and the pedigree are described in detail in Nokelainen et al. [71] and De Pasqual et al. [17]. In the rearing protocol, larvae are fed with dandelion (*Taraxacum spp*.) collected from the wild without disinfection or antibiotic supplementation. Here we modified the rearing protocol as follows. Immediately after hatching and before being given any food, we collected larvae to assess the bacteria in newly hatched larvae. Newly hatched larvae are too small for dissection (~ 2 mm), and hence, the whole larva was used. The larvae were surface sterilized to exclude microbial contamination from the environment. We cut the filter from a 1-ml filter tip and placed it inside a 1.5-ml tube. We pooled four larvae into a sample on the filter and added 450 μl of autoclaved double-distilled water (AddH_2_O), creating a whirlpool with a 1-ml filtered pipette tip for 2 min. The water was collected and the procedure was repeated three times. The collected **washing water** (Table 1) was stored at − 20 °C until DNA extraction to represent bacteria on the outside of the larva, including environmental contamination. The washed larvae were then transferred to a new 1-ml filter tip, rinsed with 450 μl of 5% sodium hypochlorite (NaOCl) solution, and centrifuged at 2000 rpm for 30 s. The rinsing process was repeated three times, after which the surface sterilized larvae were stored at − 20 °C until DNA extraction (**hatched larvae** in Table 1).

**Table 1:** Sample types included in bacterial 16S rRNA gene sequencing

Sample type	Sample type definition	n per genotype*	Total no. of samples
Hatched larvae	Surface sterilized newly hatched larvae analyzed as whole	4	8
Washing water	Water from washing newly hatched larvae	4	8
Control diet	Artificial diet	na	2
Transplant diet	Mix of control diet and frass	2	4
Frass before	Frass from larvae fed with control diet before transplantation	2	4
Frass after	Frass from larvae fed with transplant diet of the **other genotype**	WW: 3, yy: 2	5
Frass after control	Frass from larvae fed with transplant diet of the **same genotype**	2	4
Gut	Gut of adult from larvae fed with transplant diet of the **other genotype**	WW: 2, yy: 1	3
Gut control	Gut of adult from larvae fed with transplant diet of the **same genotype**	WW: 3, yy: 2	5
Abdominal fluid	Abdominal fluid of adult from larvae fed with transplant diet of the **other genotype**	WW: 10, yy: 4	14
Abdominal fluid control	Abdominal fluid of adult from larvae fed with transplant diet of the **same genotype**	WW: 8, yy: 4	12
Water control	Water used in artificial diet and sample storage	na	3

*Uneven n caused by difficulties obtaining PCR products or sample collection (i.e., abdominal fluids, guts)

The remaining larvae (374/genotype) were split into groups of 20–25 larvae and reared inside sterile petri dishes. The dishes were kept in climate chambers in a 18:6 h light:dark cycle at 21 °C during the light and 14 °C during the dark. An artificial diet was prepared consisting of 3 g agar, 32.1 g semolina, 8.58 g yeast, 8.3 g wheat germ, 1.76 g Vanderzant vitamin mix, 1.8 ml nipagin and 180 μl acetic acid in 200 ml freshly boiled AddH_2_O to minimize diet-derived bacteria. Two samples of this diet were stored at − 20 °C until DNA extraction (**control diet** in Table 1). Roughly 5 g of this diet was presented to the larvae on top of a sterilized microscope slide inside the petri dish. After 48 h, approximately 0.5 g of larval faeces, hereafter frass, was collected from the bottom of the petri dish using sterilized tweezers and stored at − 20 °C until DNA extraction (**frass before** in Table 1).

### Frass transplantation and rearing

To prepare diets for frass transplantation between genotypes, approximately 10 frass pellets from each petri dish were collected after 48 h of giving the **control diet** and pooled to obtain ~ 1 g/genotype and mixed with 50 g of the control diet. Four samples of this transplant diet (two of each genotype) were mixed with 2 ml of boiling AddH_2_O and stored at − 20 °C until DNA extraction (**transplant diet** of genotypes WW and yy in Table 1).

The larvae, all in their 3rd or 4th instar, were then divided into treatment and control groups in sterile petri dishes with 10–15 larvae of the same genotype per petri dish. The treatment group was fed the opposite genotype’s transplant diet: each petri dish of WW larvae received ~ 5 g of yy transplant diet, and each petri dish of yy larvae received ~ 5 g of WW transplant diet. The control group larvae received ~ 5 g of transplant diet of their own genotype. The petri dishes were kept in the climate cabinets in the same conditions as above. Twenty-four hours after the food was given, approximately 1 g of frass was collected from each genotype as above and stored at − 20 °C until DNA extraction (**frass after, frass after control** in Table 1). The rearing continued until all larvae pupated or died. The pupae were placed individually in 150-ml plastic containers, kept in the climate chambers in the same conditions and weighed to the nearest milligram. From a subset of the emerging adults, we dissected the gut following Moghadam et al. [67] with minor modifications. Briefly, each adult moth was placed on a sterile petri dish and its head was removed using a sterilized scalpel. A drop of AddH_2_O was placed next to the abdomen and the gastrointestinal tract (i.e., gut) including the crop, foregut, midgut, and hind gut was pulled out using sterilized forceps under a light stereoscope with a Bunsen burner next to it to reduce the risk of contamination. The dissected guts were placed individually in 30 μl of AddH_2_O and stored at − 20 °C until DNA extraction (**gut** in Table 1). Likewise, we collected the abdominal defensive secretions from the adults. We gently pressed the abdomen of live adults with sterilized tweezers until the secretion was released from the anal cavity. The secretion was collected using UV-sterilized 10-μl glass capillaries under a laminar flow, measured with a digital caliper, and placed individually in 30 μl of AddH_2_O and stored at − 20 °C until DNA extraction (**abdominal fluid** in Table 1). Finally, we took 30 μl of the AddH_2_O batch used to prepare all the samples above and stored it at − 20 °C until DNA extraction (**water control** in Table 1).

### Life histories

We followed several life-history traits of individual larvae, pupae, and adults from the different genotypes and treatments. The overall developmental rate was determined by counting the number of days elapsed from egg hatching until adult eclosion. This included the larval and pupal stages. We further analyzed the development rate within the larval stage (i.e., from egg hatching until pupation), as well as within the pupal stage (i.e., from pupation to adult eclosion). In addition, we recorded the weight of all individual pupa, and at the adult stage, we measured the volume of abdominal defensive secretions as described above.

### DNA extraction, PCR and PacBio amplicon sequencing

DNA was extracted by homogenizing the sample (larvae, frass, gut, abdominal fluid, diet) in 30 μl of water with a metal bead (∅ 2.3 mm) in a Bead Ruptor (OMNI) at speed 3.93 m/s for 2 × 30 s. After homogenization, the samples were boiled at 100 °C for 10 min and stored at -20 °C until further use. DNA quantification was performed with the Qubit BR DNA kit (ThermoFisher).

To assess bacterial diversity in the larvae, their frass, and adult moths, we amplified ~ 1550 bp of the 16S ribosomal RNA (rRNA) gene using custom primers (forward 5′-AGAGTTTGATCMTGGCTCAG-3′, reverse 5′-CCTTGTTACGACTTCACCCCAG-3′). The primers were designed using Primer3 [112] from Lepidoptera-associated 16S rRNA gene sequences downloaded from National Center for Biotechnology Information (NCBI) and aligned using ClustalW [95]. Polymerase chain reactions (PCR) were performed in a C1000 thermal cycler (Bio-Rad) using 5 μl of DNA template, 1 × Platinum SuperFI Mastermix (ThermoFisher), 1 × enhancer buffer, 0.5 μM of each primer, and 0.5 mM MgCl_2_ in reaction volume of 20 μl. The cycling conditions were as follows: 98 °C for 30 s, 40 cycles of 98 °C for 10 s, 49 °C for 10 s, 72 °C for 1 min, and a final elongation of 5 min at 72 °C. The PCR products were run in 3% agarose gels, and the bands were excised using gel cutting tips (Axygen) and purified by centrifuging through 1-ml filter tips at 6000 rpm for 15 min. DNA concentration of the purified PCR products was measured using PicoGreen dsDNA Assay Kit (ThermoFisher), and a sequencing library was prepared according to the PacBio multiplexed amplicon library preparation protocol. The samples were barcoded and sequenced in a PacBio Sequel at the Novogene sequencing laboratories. In addition, a mock community (ZymoBIOMICS Microbial Community DNA Standard, Zymo Research) and the water control (i.e., negative control) were amplified and sequenced with each library.

### Sequence processing and quality control

Sequence reads were processed with PacBio tools distributed in Bioconda. PacBio subreads were combined into consensus sequences with ccs in package pbccs (v.6.0.0) with the default settings. The consensus sequences (797,984 reads) were demultiplexed based on barcodes with lima (v. 2.0.0) with the settings -peak-guess, -different- -ccs, -min-length 1440, -max-input-length 1580, -min-end-score 26, and -split-bam-named. The resulting bam files were converted into fastq with bam2fastq in the package bam2fastx (v. 1.3.1). Sequences were submitted to the National Center for Biotechnology Information Sequence Read Archive under accession code PRJNA804133.

The reads were processed further and amplicon sequence variants (ASVs) inferred in DADA2 (v. 1.16.0, [8] following guidelines for PacBio data [9], https://benjjneb.github.io/LRASManuscript/LRASms_fecal.html) in R (v. 4.0.4, [84] and RStudio (v. 1.4.1106). Primers were removed with the command removePrimers. Reads were filtered with the command filterAndTrim and the settings minQ = 3, minLen = 1300, maxLen = 1600, maxN = 0, and maxEE = 2. The reads were dereplicated and denoised using PacBio-specific error estimation function. Chimeras were removed from the denoised reads with the command removeBimeraDenovo and setting minFoldParentOverAbundance = 3.5. Taxonomy was assigned against the Silva database (v. 138.1, [83]. The ASV data was imported into phyloseq (v. 1.32.0, [64], and ASVs assigned to chloroplasts or mitochondria or not assigned to Bacteria were removed. Potential contaminants were examined based on ASVs in water control and washing water samples (Table 1) with the package decontam based on frequency (threshold 0.2), prevalence (threshold 0.5), and inspection of frequency vs. DNA concentration plots [16], and none were detected. This resulted in 226 ASVs (when excluding ASVs in the mock community controls) and on average 6898 reads per sample (in total 565,705 reads).

### Statistical analyses and phylogenetic diversity measures

All analyses were carried out in R (v. 4.0.4) through RStudio. The package ggplot2 [122] was used for generating plots. To examine the effect of the frass transplantation on the life histories of the genotypes, we implemented a Kruskal–Wallis one-way analysis of variance (ANOVA) followed by pairwise Dunn’s tests to the traits measured. All tests’ significance values were corrected for multiple comparisons. This non-parametric approach was chosen because the samples violate parametric assumptions of normality and/or equality of variance (Additional file 1: Fig. S2).

For comparing bacterial diversity and community composition, the ASV table was rarefied to the median number of reads (6915 reads) with the function rrarefy in vegan (v. 2.5.7, [74]. If a sample had fewer reads than the median, all its reads were included. Then, the ASV table was standardized to relative abundances. Sequences of the ASVs were aligned and a phylogenetic tree was constructed using RAxML (model GRT + gamma, [101]) on the SILVA ACT server [82]. Faith’s phylogenetic diversity (PD) and ASV richness were determined in picante (v. 1.8.2, [49]. The phylogenetic tree was converted into a phylogenetic distance matrix with the function cophenetic. Measures of phylogenetic relatedness among communities (phylogenetic beta diversity) were calculated in picante as mean pairwise distance (MPD, [120], function comdist, abundance weighted) and mean nearest taxon distance (MNTD, function comdistnt, abundance weighted). MPD emphasizes the clustering of basal clades in the phylogenetic tree, whereas MNTD emphasizes patterns closer to the tips of the tree. These values were used as distance measures in non-metric multidimensional scaling (NMDS) with function metaMDS in vegan and permutational multivariate analysis of variance (PERMANOVA) with function adonis2 in vegan [2].

Separate phylogenetic trees of *Erysipelotrichaceae* and *Enterococcus* ASVs were constructed by aligning the sequences and selected reference sequences (described strains and similar environmental sequences identified by Blast searches) with SINA v. 1.2.11 on the SILVA ACT server and inferring a maximum likelihood tree with RAxML (model GTR + gamma, [101]) in QIIME2 (v. 2021.8.0, [4].

We used the same phylogenetic relatedness measures as above to assess phylogenetic clustering of bacterial communities against null models across sample types. This analysis aims to determine whether bacterial community assembly and turnover across life stages are driven by niche-based (i.e., environmental filtering) or stochastic processes [104]. For example, if gut conditions favour the proliferation of specific bacterial taxa, it would be shown as phylogenetic clustering. Net relatedness index (NRI) was calculated as the standardized effect size of MPD (function ses.mpd in picante, abundance weighted) multiplied by -1. Nearest taxon index (NTI) was calculated as the standardized effect size of MNTD (function ses.mntd, abundance weighted) multiplied by -1. Positive values of NRI and NTI indicate phylogenetic clustering (taxa are more closely related than by chance), whereas negative values indicate phylogenetic overdispersion (taxa are less closely related than by chance). Values of NRI and NTI differing from 0 and thus showing more clustering by chance were identified by Welch’s t-test (function t.test). To compare mechanisms of phylogenetic turnover along life history and experimental stages, we calculated βNTI values for pairs of communities according to Stegen et al. [104] and https://github.com/stegen/Stegen_etal_ISME_2013. βNTI > 2 indicates significantly higher community turnover than by chance driven by deterministic selection [104, 105]. βNTI between -2 and 2 indicates community assembly driven by stochastic processes. βNTI < -2 indicates less community turnover than by chance.

## Results

### Life-history effects of frass transplantation

**Fig. 1:**
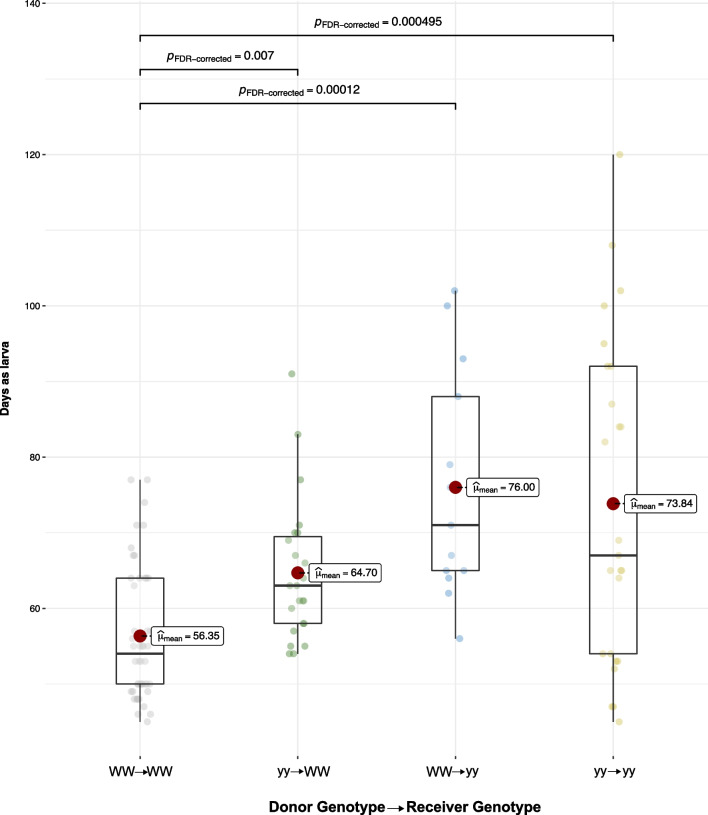
Days as larvae of *A. plantaginis* in reciprocal frass transplantation between genotypes WW and yy and in control transplantations within the genotypes

Overall developmental time (i.e., from egg hatching to adult eclosion) differed between the controls of genotypes WW and yy (Additional file 1: Fig. S3), as in the stock selection lines (Additional file 1: Fig. S1). This difference was mainly due to the faster growth of WW individuals at the larval stage (Fig. 1). The length of the pupal stage did not differ between the genotypes (Kruskal–Wallis one-way ANOVA statistic = 7.74, *P* > 0.05) (data not shown). When WW larvae received the frass transplant from genotype yy, they grew slower than the control WW larvae that received WW frass (Fig. 1, Additional file 1: Fig. S3). The opposite was not observed: WW frass transplant did not affect the growth of yy larvae.

Pupal weight differed with genotype in the controls, with WW pupae being lighter than yy pupae (Additional file 1: Fig. S4). However, we observed no differences in pupal weight between the controls and the pupae that received the frass transplant of the other genotype. This suggests that the frass transplant did not affect pupal weight. Transplantation also did not affect pupal mortality (controls WW 10%, yy 12%; treatments yy to WW 7%, WW to yy 15%).

**Fig. 2:**
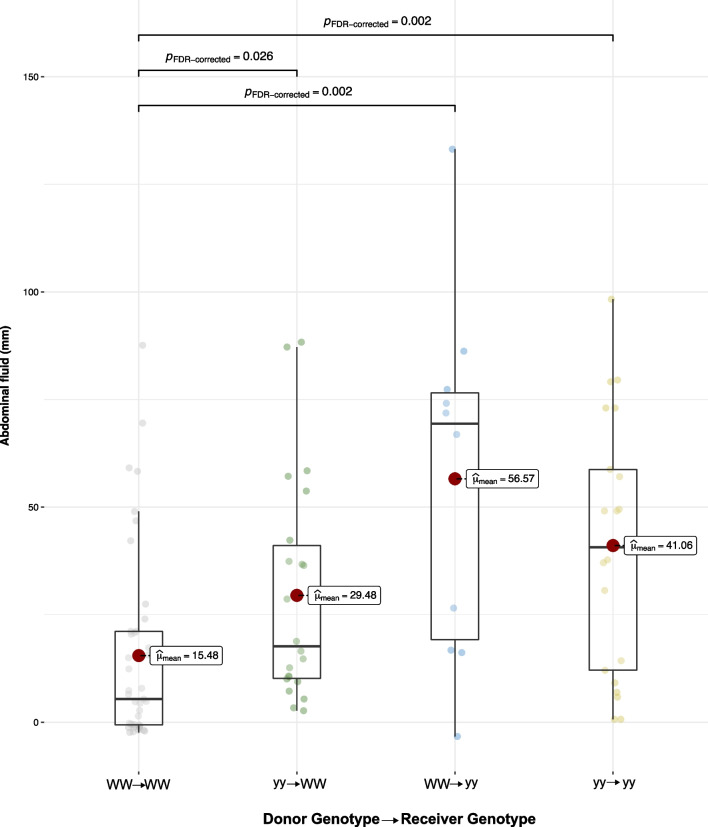
Secretion of defensive abdominal fluids of *A. plantaginis* adults in reciprocal frass transplantation between genotypes WW and yy and in control transplantations within the genotypes

The adults of genotype WW secreted a smaller volume of defensive abdominal fluids than the adults of yy in the controls. Transplantation with yy frass significantly increased abdominal fluid secretion in WW adults (Fig. 2). As in the case of growth rate, this could point to greater susceptibility or adaptability of the WW genotype to the transplantation and the presence of foreign bacteria. Larger volumes in defensive secretions were also observed in the yy genotype transplanted with WW frass. However, this difference was not significant relative to the yy controls, likely because of the unbalanced number of samples. Overall, it can be suggested that frass transplantation generally increased the volume of defensive secretions.

### Bacterial community composition and diversity with genotype, life stage and frass transplant

**Fig. 3:**
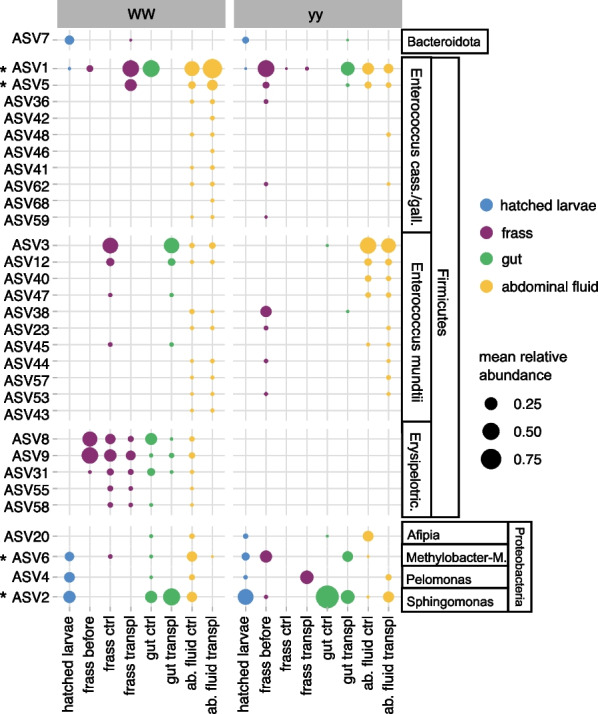
Distribution of bacterial 16S rRNA gene amplicon sequence variants (ASVs) in *A. plantaginis* genotypes WW and yy with life stage in frass transplantations between genotypes (transpl) and in control transplantation within genotypes (ctrl). Only ASVs occurring in more than two samples are shown. * marks ASVs with > 50% prevalence across the samples. Replicate samples within the same sample type and genotype have been merged and the mean relative abundance of ASVs is shown. Cass. stands for *casseliflavus*, gall. for *gallinarum*, Erysipelotric. for *Erysipelotrichaceae*, Methylobacter-M. for *Methylobacter-Methylorubrum*, and ab. fluid for abdominal fluid

Bacteria detected in newly hatched larvae showed no difference in phylogenetic diversity or ASV richness between the genotypes (Additional file 1: Fig. S5). Compared to the newly hatched larvae, larval frass before transplantation had lower phylogenetic diversity (Additional file 1: Fig. S5), which indicated that frass contained a reduced set of bacteria. However, the frass bacteria were not solely a subset of the bacteria detected in newly hatched larvae. Frass before transplantation shared only one out of five ASVs (genotype WW) or three out of 11 ASVs (genotype yy) with the newly hatched larvae (Fig. 3).

**Fig. 4:**
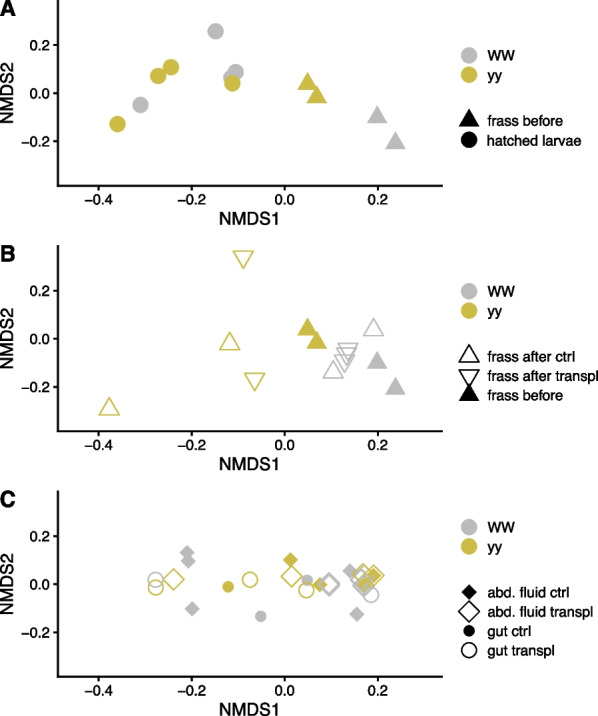
Non-metric multidimensional scaling (NMDS) plots of bacterial community in *A. plantaginis* genotypes WW and yy based on phylogenetic distances (MNTD, mean nearest taxon distance). **A** Newly hatched larvae and frass before transplantation, **B** frass before and after transplantation in controls within genotypes (ctrl) and between genotypes WW and yy (transpl), **C** gut and abdominal fluid (abd. fluid). Panels A, B and C are based on the same ordination but plotted separately. Stress = 0.15

**Fig. 5:**
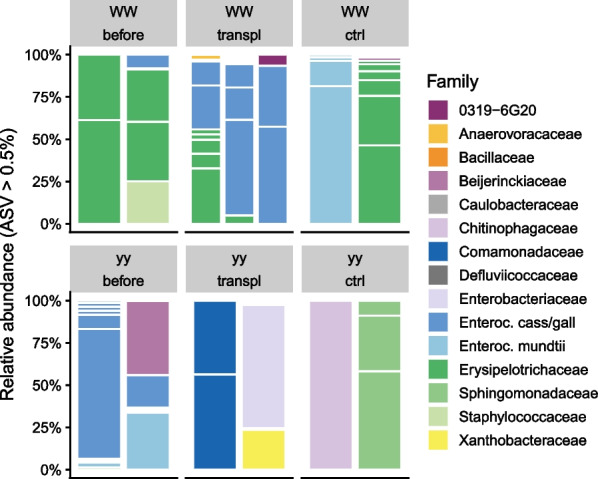
Taxonomic distribution of bacterial 16S rRNA gene amplicon sequence variants (ASVs) at family level in frass before and after transplantation within genotypes WW and yy (ctrl) and between genotypes WW and yy (transpl). White horizontal lines separate ASVs. Each ASV with relative abundance above 0.5% is shown as its own section in the columns. Family ‘0319-6G20’ belongs to phylum *Bdellovibrionota*, class *Oligoflexia*. Enteroc. stands for *Enterococcus*, and cass/gall for *casseliflavus*/*gallinarum*

Unlike in newly hatched larvae, the bacterial community in frass differed between the genotypes (Fig. 4, Additional file 1: Fig. S6; PERMANOVA hatched larvae R^2^ = 0.27, *P* = 0.24; frass R^2^ = 0.38, *P* = 0.001). Frass of genotype WW collected before transplantation had lower ASV richness and phylogenetic diversity than yy frass (Additional file 1: Fig. S5). Bacteria in WW frass were dominated by *Erysipelotrichaceae* (*Firmicutes*; Fig. 5), which were only detected in genotype WW: in addition to larval frass in WW adult gut and abdominal fluids (Fig. 3). These *Erysipelotrichaceae* belong to a novel cluster with no described species that includes uncultured bacteria detected in termite gut (Additional file 1: Fig. S7; [40, 65]. Frass of genotype yy was instead dominated by *Enterococcaceae* (*Firmicutes*,Fig. 5). These ASVs included ASV1 and ASV5 (Fig. 3) that occurred across the genotypes in frass, adult gut, and abdominal fluids and had 100% sequence similarity to *Enterococcus gallinarum* and *E. casseliflavus* strains from Lepidoptera (Additional file 1: Fig. S8; [14, 15]. These *Enterococcaceae* ASVs were among the only four common core ASVs detected in one or more sample types with 50% prevalence (Fig. 3).

When WW larvae received the yy frass transplant, their frass retained the WW-specific *Erysipelotrichaceae*. Notably, WW frass also gained or showed an increased relative abundance of the two common *Enterococcaceae* ASVs detected in yy frass (ASV1, ASV5) (Fig. 5). The prominence of these ASVs in WW frass after transplantation suggests they may have originated from the yy frass transplant. *Enterococcus* ASVs became more prevalent also in one of the WW controls that received WW frass, but these ASVs represented a different cluster within *Enterococcus* not detected in yy frass (ASV3, ASV12, 99–100% sequence similarity to *E. mundtii* strains, Additional file 1: Fig. S9). Bacteria in yy frass, on the other hand, changed almost completely both with WW frass transplant and in the controls that received genotype yy’s own frass (Figs. 4B, 5). After the transplant, yy frass bacteria consisted of sporadic ASVs representing bacterial groups detected in the control diet and transplant diet fed to the larvae (Fig. 5, Additional file 1: Fig. S9). Therefore, we found no evidence pointing to the transfer of WW frass bacteria to genotype yy. Variation of bacterial community in adult gut and abdominal fluid could not be linked to the transplant treatment or to genotype, partly due to large variation among individuals (Fig. 4C, Additional file 1: Fig. S10). The ASVs affiliated with *E. casseliflavus/gallinarum* and *E. mundtii* occurred in adult gut with no consistent pattern. In adult abdominal fluids, both *Enterococcus* types were present in both genotypes, but *E. casseliflavus/gallinarum* ASVs dominated in genotype WW and *E. mundtii* type in genotype yy (Additional file 1: Fig. S10).

### Phylogenetic clustering and turnover of bacteria with life stage and genotype

**Fig. 6:**
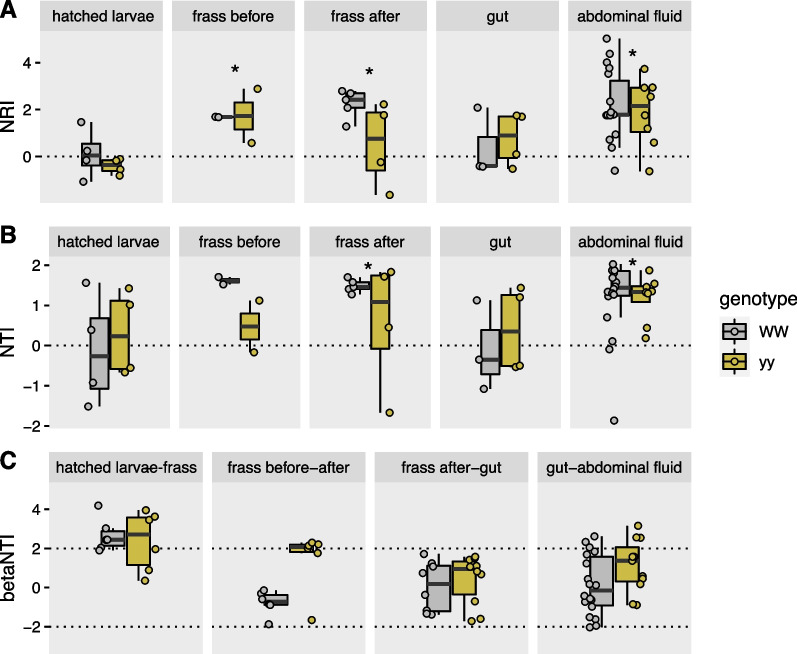
**A** Net relatedness index (NRI) and **B** nearest taxon index (NTI) with life stage and genotype (WW, yy) **C** and beta nearest taxon index (βNTI) for bacterial community data between different stages. NRI > 0 and NTI > 0 (indicated in A and B by * when significant difference from 0 in t-test) show greater phylogenetic clustering than by chance. βNTI > 2 indicates significantly higher community turnover than by chance driven by deterministic selection, whereas − 2 < βNTI > 2 suggests stochastic community assembly

We used phylogenetic clustering and turnover measures to identify potential ecological mechanisms structuring the bacterial community with life stage and genotype. Bacterial communities in larval frass before and after transplantation and in adult abdominal fluid showed higher phylogenetic clustering than by chance (NRI, NTI > 0, Fig. 6A, B), which suggests selection or environmental filtering of specific bacterial lineages. In newly hatched larvae and adult gut, NRI and NTI did not differ from 0, and thus no evidence of selection of specific lineages was detected. Only frass before and after transplant showed potential differences in the extent of clustering with genotype. When looking at phylogenetic turnover in transitions between life stages, bacterial community turnover was largest in the transition from newly hatched larvae to frass (Fig. 6C). βNTI values of > 2 indicated deterministic selection in this community shift. Community dynamics of the other transitions appeared more driven by stochastic processes (− 2 < βNTI > 2). Difference in βNTI values between the genotypes in frass suggests that genotype affected the dynamics and community assembly of the frass bacterial community.

## Discussion

Bacteria in insect hosts have been connected to fitness and development [21, 32] and even to driving changes in host allele frequencies [89]. Many insects show highly species-specific microbiome composition, but Lepidoptera are considered to deviate from this pattern of insect phylosymbiosis [59], but see [33]. Consequently, the mechanisms of bacterial community assembly and the role of bacteria in key traits such as growth and defensive mechanisms of Lepidoptera remain unresolved [35, 77]. Here, we show that a reciprocal frass transplantation between a fast-growing and a slow-growing genotype of the wood tiger moth reversed the growth rate, but only in the fast-growing genotype, also increasing the volume of defensive secretions. The host bacterial community had components that (1) were genotype specific, (2) passed strong environmental filtering in the gut, and (3) were retained through life stages. Together, these findings suggest that whether the microbiome influences Lepidoptera growth may depend on genotype-specific ability of the host to gain and retain specific bacteria.

The life-history traits of the fast-growing genotype WW were more susceptible to frass transplantation than those of the slow-growing genotype yy. In addition to growth rate, the white and yellow colour morphs that the genotypes represent differ in their immune response mechanisms. The yellow morph that the genotype yy represents has more effective lytic activity in its haemolymph [70]. This stronger innate immune response against bacterial invaders could partly explain why the slow-growing genotype yy was more resistant to frass transplantation than the WW genotype. The immune system has been proposed as one of the mechanisms driving microbiome differences between hosts with different genetic backgrounds [24, 51, 100]. Furthermore, the costs of this stronger immune defence in genotype yy could be reflected in slower growth [3, 6, 96].

Effective defence against bacterial invaders in the yy genotype could have prevented it from acquiring bacteria from the frass transplant or the environment that are potentially beneficial for growth in the fast-growing genotype WW. Alternatively, or in addition, the chemical or physiological environment in the gut of genotype WW may favour the establishment of different bacteria [51]. We detected *Erysipelotrichaceae* only in the fast-growing genotype WW, and their relative abundance decreased together with growth rate after yy frass transplantation. Transplantation increased *Enterococcus* ASVs seen in yy frass, which could imply the transfer of these bacteria to the WW genotype. We detected no transfer of bacteria from WW to yy. One limitation here is that we cannot rule out changes in bacterial community composition in frass after it was excreted and before it was collected. Nevertheless, WW-specific *Erysipelotrichaceae* (increasing growth), yy-originating *Enterococcus* (constraining growth) or the balance between these groups could presumably be a bacterial component associated with larval growth.

*Erysipelotrichaceae,* which can be considered genotype-specific core taxa here, commonly occur in insect guts [108]. For example, they occur in dung beetles depending on genus and diet [19], and in Lepidoptera depending on growth environment [28] and season [38]. The family *Erysipelotrichaceae* consists of anaerobic or aerotolerant bacteria with fermentative metabolism [108]. However, the ASVs we detected are too distant from any described strains (Additional file 1: Fig. S7) to allow more detailed speculation on their metabolism. In vertebrates, a relative increase of *Erysipelotrichaceae* has been connected to dietary fat, weight gain, metabolic disorders, and high feed-to-weight conversion rate [47, 102, 111, 125]. Congruently, their decrease has been linked to reduced growth [5]. These findings show *Erysipelotrichaceae* as a responsive member of the gut microbiota with connections to lipid metabolism and growth. Here, we linked *Erysipelotrichaceae* to lepidopteran larval growth rate when the genetic background allowed the establishment of these bacteria in the gut. Characterization of isolates or genomes of this distinct novel cluster of *Erysipelotrichaceae* is required to establish potential mechanisms for how these bacteria affect growth, directly or indirectly.

The other scenario that our results put forward is that specific members of *Enterococcus* could have an impact in reducing larval growth rate. Enterococci are lactic acid bacteria well adapted to survival in the harsh gut environment by evading host defences [22, 63] and commonly found in Lepidoptera throughout their life cycle [13, 29, 36, 109]. The specific enterococci we detected in connection to slower growth clustered with *E. casseliflavus* and *E. gallinarum*, which can be the dominant bacteria in Lepidoptera [44, 60, 93, 106]. These enterococci have been suggested to potentially play beneficial roles in larvae, such as detoxifying diet plant compounds [115] and degrading cellulose and proteins [15, 81], which may not align with reduced growth. On the other hand, strains of *E. casseliflavus* have also been reported to be pathogenic to larvae [92, 110], though not in all cases [76]. The larvae in our experiment showed no signs of pathogenic effects, and the only negative outcome we can connect is the presence of *E. casseliflavus/gallinarum* with slower larval growth. In the control transplant of the faster-growing WW genotype, we instead detected enterococci that grouped with *E. mundtii*. This proposed lepidopteran symbiont has been reported to be antagonistic against potential pathogens, including *E. casseliflavus,* by producing an antimicrobial peptide and to appear later in larval development than *E. casseliflavus* [43, 45, 92]. Overall, our results suggest that the establishment and dynamics of these two clusters of enterococci (*E. casseliflavus/gallinarum* vs. *E. mundtii*) can be influenced by host genotype with potential effects on larval growth.

In adults of the wood tiger moth which, in contrast to larvae, are short-lived and do not feed, we detected both *E. mundtii* and *E. casseliflavus/gallinarum* ASVs across genotypes and treatments. This co-occurrence implies that different mechanisms could control their community dynamics in adults vs larvae. If these dynamics are further sensitive to the host's genetic background, as our results suggest, the specific functional roles of these enterococci are most likely highly context dependent. It is possible that under some conditions, they form a host-adapted core microbiome [85, 94], which is often considered lacking in Lepidoptera. The context-dependency would, however, suggest that any effects of these enterococci on growth are causal role functions depending on the presence or absence of the microbe, rather than selected effect functions driven by evolutionary mechanisms and consistent maintenance of the microbe in the host population [50]. Further manipulative studies targeted at enriching or depleting such taxa are needed to confirm this notion. In addition, it should be taken into account that frass transplants contain not only bacteria but also other microbes such as viruses and fungi, which could influence the bacterial dynamics and life-history outcomes.

Our results agree with previous findings of low bacterial diversity in Lepidoptera and a lack of a consistent core microbiome with striking variability among individuals [44, 61, 66]. The artificial diet we fed the larvae to minimize the presence of non-frass microbes most likely led to even lower diversity than in wild-collected or plant-fed larvae. This low bacterial load could also accentuate stochastic processes such as ecological drift and priority effects more than in hosts with more diverse and stable microbiomes, leading to high inter-individual variation [10, 51]. Yet, against this low and variable background, the results also support previous notions that specific taxa have an advantage in the harsh conditions of the lepidopteran larval gut [106]. Taxa characteristic to a genotype (*Erysipelotrichaceae*) or common in Lepidoptera (*E. casseliflavus/gallinarum, E. mundtii*), which were not detected or barely detected among the diverse bacteria of the newly hatched larvae, became dominant in larval frass via non-random selection and were retained in adults (Figs. 3, 6). Moreover, the filtering process in the larvae appeared to be largely genotype specific. In the adults, the deterministic and genotype-specific patterns seen in the larvae were attenuated, which may reflect the role of the adult stage in the life history of the wood tiger moth as a very short reproductive stage (5–7 days). A caveat here is that our set of adult gut samples is relatively small due to difficulties in dissecting them. Since the adults do not feed, functional guts are not necessary, and their gut structures are the remnants of the larval gut that are partly absorbed during metamorphosis. Nevertheless, our results provide a rare view of lepidopteran bacterial community dynamics through life stages in a species that does not feed as an adult. Butterflies that do feed as adults display relatively more consistent bacterial community composition in adults compared to larvae [33]. In the non-feeding wood tiger moth, bacteria in both whole larvae and adults varied greatly, and passage through the larval gut to frass was the most selective step.

The frass transplantation impacted the volume of abdominal secretions in the same way as the growth rate of the genotypes. The WW genotype significantly increased its defensive secretions, whereas the expected opposite decrease in the yy genotype was not observed (Fig. 2). A recent study found no differences in the volume of abdominal secretions of wild-caught or laboratory-reared white and yellow wood tiger moths [57]. Here, we observed larger volumes in the yy control genotype relative to the WW control genotype, and a general increase in both genotypes after frass transplantation. Different diets may partly explain the discrepancies between the studies. For instance, laboratory-reared moths were fed dandelion (*Taraxacum* spp.) in the previous study, whereas an artificial diet was used here. Likewise, the larval diet of wild-caught adults is unknown. Hence, it is difficult to draw comparisons between absolute volumes of defensive secretions. However, it can be inferred that diet is not a major contributor to the defensive secretions, because the frass transplantation increased the volume of defensive secretions in both genotypes, which fed on the same artificial diet mixed with frass of the other genotype.

Differences in antipredator efficacy have also been reported, where the abdominal secretions of yellow moths are more deterrent against ants than secretions from white individuals [87]. The exact chemical composition of the abdominal fluids has not been characterized. However, the wood tiger moth can sequester hepatoxic organic compounds, such as pyrrolizidine alkaloids (PAs), from its diet [123], and these compounds have been detected in the abdominal secretions of wild-caught adults (Winters et al., unpublished). PAs are well-known for providing protection to plants against herbivores, which in turn can host bacterial associates with detoxifying abilities [46, 107]. Some bacteria have even been suggested to synthesize PAs, as inferred by the presence of alkaloid biosynthesis gene clusters [91]. Manipulative experiments are needed to study PA sequestration-detoxification processes in the wood tiger moth. The great diversity of bacteria found in the abdominal secretions holds great potential to help elucidate the mechanism underlying these processes and to better understand differences in protection between the colour morphs.

The great and largely unexplained variation of bacteria among individuals in the defensive secretions represents an example of how the drivers of bacterial community composition can be decoupled in larvae vs. adults [34]. We have previously shown that the wood tiger moth's pre- and post-metamorphosis life stages are only partly decoupled [26]. Our across-life stage analysis provides additional evidence for this partial de-coupling. For instance, the genotype-specificity of *Erysipelotrichaceae* remained in the abdominal fluids, which showed that their bacterial community was not completely uncoupled from the previous life stages. A curious observation was a higher diversity of *Enterococcus* ASVs in the abdominal fluids compared to larvae, frass or gut. Together with the signs of phylogenetic clustering in the fluids, this finding suggests that distinct and partly unidentified drivers govern the bacterial community dynamics in the adult’s defensive secretions vs. in the larvae.

## Conclusions

We show for the first time that genotype-specific life-history traits in a lepidopteran host can be reversed with a reciprocal frass transplantation. Our results help clarify bacterial community assembly in Lepidoptera at different life-stages and relate the bacterial community composition to the genotype and growth of the wood tiger moth. With nearly full-length 16S rRNA gene amplicons, we were able to discern the dynamics of closely related enterococci and recovered a novel cluster of insect-associated, genotype-specific *Erysipelotrichaceae*.

Our findings indicate that an insect host can fine-tune its bacterial community in a genotype-specific manner even against a highly variable bacterial community background with stochastic community assembly. The strong selection of bacteria in larval frass appeared to be relaxed in adults, which shows that previous findings of consistent bacterial community in adult Lepidoptera [33] may not apply to species that do not feed as adults. Thus, not only life stage but also species-specific adult feeding habits and host genotype can influence bacterial community assembly contributing to the high variation and inconsistencies observed in Lepidoptera microbiomes. Overall, our results suggest that the digestive processes of slow- and fast-growing host genotypes differ in filtering or retaining specific bacterial groups. Looking deeper into the functional, chemical and genomic background of these differences could reveal the molecular and ecological mechanism of the filtering.

## Supplementary Information

**Additional file 1:** Figures S1–S10.

## Data Availability

The sequence data are available in the National Center for Biotechnology Information Sequence Read Archive under accession code PRJNA804133. R scripts and data files for microbiome analysis are available at https://github.com/helijuottonen/mothtransplant. R scripts and data files for life history analysis will be available at https://github.com/Juan-Galarza?tab=repositories.

## References

[1] Allegretti JR, Mullish BH, Kelly C, Fischer M (2019). The evolution of the use of faecal microbiota transplantation and emerging therapeutic indications. The Lancet.

[2] Anderson MJ (2001). A new method for non-parametric multivariate analysis of variance. Aust Ecol.

[3] Ardia DR, Gantz JE, Schneider BC, Strebel S (2012). Costs of immunity in insects: an induced immune response increases metabolic rate and decreases antimicrobial activity: energetic costs of immunity. Funct Ecol.

[4] Bolyen E, Rideout JR, Dillon MR, Bokulich NA, Abnet CC, Al-Ghalith GA, Alexander H (2019). Reproducible, interactive, scalable and extensible microbiome data science using QIIME 2. Nat Biotechnol.

[5] Bao Z, Zhao Y, Wu A, Lou Z, Lu H, Yu Q, Fu Z, Jin Y (2020). Sub-chronic carbendazim exposure induces hepatic glycolipid metabolism disorder accompanied by gut microbiota dysbiosis in adult zebrafish (Daino rerio). Sci Total Environ.

[6] Boots M, Begon M (1993). Trade-offs with resistance to a granulosis virus in the Indian meal moth, examined by a laboratory evolution experiment. Funct Ecol.

[7] Broderick NA, Raffa KF, Goodman RM, Handelsman J (2004). Census of the bacterial community of the gypsy moth larval midgut by using culturing and culture-independent methods. Appl Environ Microbiol.

[8] Callahan BJ, McMurdie PJ, Rosen MJ, Han AW, Johnson AJA, Holmes SP (2016). DADA2: high-resolution sample inference from Illumina amplicon data. Nat Methods.

[9] Callahan BJ, Wong J, Heiner C, Oh S, Theriot CM, Gulati AS, McGill SK, Dougherty MK (2019). High-throughput amplicon sequencing of the full-length 16S rRNA gene with single-nucleotide resolution. Nucleic Acids Res.

[10] Chase JM, Myers JA (2011). Disentangling the importance of ecological niches from stochastic processes across scales. Philos Trans R Soc B: Biol Sci.

[11] Chaston JM, Dobson AJ, Newell PD, Douglas AE (2016). Host genetic control of the microbiota mediates the Drosophila nutritional phenotype. Appl Environ Microbiol.

[12] Chaturvedi S, Rego A, Lucas LK, Gompert Z (2017). Sources of variation in the gut microbial community of *Lycaeides melissa* caterpillars. Sci Rep.

[13] Chen B, Teh B-S, Sun C, Hu S, Lu X, Boland W, Shao Y (2016). Biodiversity and activity of the gut microbiota across the life history of the insect herbivore *Spodoptera littoralis*. Sci Rep.

[14] Chung J, Jeong H, Ryu C-M (2018). Complete genome sequences of *Enterobacter cancerogenus* CR-Eb1 and *Enterococcus* sp. strain CR-Ec1, isolated from the larval gut of the greater wax moth, *Galleria mellonella*. Genome Announc.

[15] Dantur KI, Enrique R, Welin B, Castagnaro AP (2015). Isolation of cellulolytic bacteria from the intestine of *Diatraea saccharalis* larvae and evaluation of their capacity to degrade sugarcane biomass. AMB Express.

[16] Davis NM, Proctor DM, Holmes SP, Relman DA, Callahan BJ (2018). Simple statistical identification and removal of contaminant sequences in marker-gene and metagenomics data. Microbiome.

[17] De Pasqual C, Suisto K, Kirvesoja J, Gordon S, Ketola T, Mappes J (2022). Heterozygote advantage and pleiotropy contribute to intraspecific color trait variability. Evolution.

[18] Douglas AE (2015). Multiorganismal insects: diversity and function of resident microorganisms. Annu Rev Entomol.

[19] Ebert KM, Arnold WG, Ebert PR, Merritt DJ (2021). Hindgut microbiota reflects different digestive strategies in dung beetles (Coleoptera: Scarabaeidae: Scarabaeinae). Appl Environ Microbiol.

[20] Engel P, Moran NA (2013). The gut microbiota of insects: diversity in structure and function. FEMS Microbiol Rev.

[21] Feldhaar H, Gross R (2009). Insects as hosts for mutualistic bacteria. Int J Med Microbiol.

[22] Fiore E, Van Tyne D, Gilmore MS (2019). Pathogenicity of enterococci. Microbiol Spect.

[23] Foster KR, Schluter J, Coyte KZ, Rakoff-Nahoum S (2017). The evolution of the host microbiome as an ecosystem on a leash. Nature.

[24] Franzenburg S, Walter J, Künzel S, Wang J, Baines JF, Bosch TCG, Fraune S (2013). Distinct antimicrobial peptide expression determines host species-specific bacterial associations. Proc Natl Acad Sci.

[25] Galarza JA, Nokelainen O, Ashrafi R, Hegna RH, Mappes J (2014). Temporal relationship between genetic and warning signal variation in the aposematic wood tiger moth (*Parasemia plantaginis*). Mol Ecol.

[26] Galarza JA, Dhaygude K, Ghaedi B, Suisto K, Valkonen J, Mappes J (2019). Evaluating responses to temperature during pre-metamorphosis and carry-over effects at post-metamorphosis in the wood tiger moth (*Arctia plantaginis*). Philos Trans R Soc B: Biol Sci.

[27] Galarza JA, Murphy L, Mappes J (2021). Antibiotics accelerate growth at the expense of immunity. Proc R Soc B: Biol Sci.

[28] Gomes AFF, Omoto C, Cônsoli FL (2020). Gut bacteria of field-collected larvae of *Spodoptera frugiperda* undergo selection and are more diverse and active in metabolizing multiple insecticides than laboratory-selected resistant strains. J Pest Sci.

[29] González-Serrano F, Pérez-Cobas AE, Rosas T, Baixeras J, Latorre A, Moya A (2020). The gut microbiota composition of the moth *Brithys crini* reflects insect metamorphosis. Microb Ecol.

[30] Gordon SP, Kokko H, Rojas B, Nokelainen O, Mappes J (2015). Colour polymorphism torn apart by opposing positive frequency-dependent selection, yet maintained in space. J Anim Ecol.

[31] Gould AL, Zhang V, Lamberti L, Jones EW, Obadia B, Korasidis N, Gavryushkin A, Carlson JM, Beerenwinkel N, Ludington WB (2018). Microbiome interactions shape host fitness. Proc Natl Acad Sci.

[32] Gupta A, Nair S (2020). Dynamics of insect–microbiome interaction influence host and microbial symbiont. Front Microbiol.

[33] Hammer TJ, Dickerson JC, McMillan WO, Fierer N (2020). *Heliconius* butterflies host characteristic and phylogenetically structured adult-stage microbiomes. Appl Environ Microbiol.

[34] Hammer TJ, Moran NA (2019). Links between metamorphosis and symbiosis in holometabolous insects. Philos Trans R Soc B: Biol Sci.

[35] Hammer TJ, Janzen DH, Hallwachs W, Jaffe SP, Fierer N (2017). Caterpillars lack a resident gut microbiome. Proc Natl Acad Sci.

[36] Hammer TJ, McMillan WO, Fierer N (2014). Metamorphosis of a butterfly-associated bacterial community. PLoS ONE.

[37] Hegna RH, Galarza JA, Mappes J (2015). Global phylogeography and geographical variation in warning coloration of the wood tiger moth (*Parasemia plantaginis*). J Biogeogr.

[38] Higuita Palacio MF, Montoya OI, Saldamando CI, García-Bonilla E, Junca H, Cadavid-Restrepo GE, Moreno-Herrera CX (2021). Dry and rainy seasons significantly alter the gut microbiome composition and reveal a key *Enterococcus* sp. (Lactobacillales: Enterococcaceae) core component in Spodoptera frugiperda (Lepidoptera: Noctuidae) corn strain from Northwestern Colombia. J Insect Sci.

[39] Holt JF, Kiedrowski MR, Frank KL, Du J, Guan C, Broderick NA, Dunny GM, Handelsman J (2015). *Enterococcus faecalis* 6-phosphogluconolactonase is required for both commensal and pathogenic interactions with *Manduca sexta*. Infect Immun.

[40] Hongoh Y, Deevong P, Inoue T, Moriya S, Trakulnaleamsai S, Ohkuma M, Vongkaluang C, Noparatnaraporn N, Kudo T (2005). Intra- and interspecific comparisons of bacterial diversity and community structure support coevolution of gut microbiota and termite host. Appl Environ Microbiol.

[41] Hosokawa T, Ishii Y, Nikoh N, Fujie M, Satoh N, Fukatsu T (2016). Obligate bacterial mutualists evolving from environmental bacteria in natural insect populations. Nat Microbiol.

[42] Jahnes BC, Herrmann M, Sabree ZL (2019). Conspecific coprophagy stimulates normal development in a germ-free model invertebrate. PeerJ.

[43] Jarosz J (1979). Gut flora of *Galleria mellonella* suppressing ingested bacteria. J Invertebr Pathol.

[44] Jones AG, Mason CJ, Felton GW, Hoover K (2019). Host plant and population source drive diversity of microbial gut communities in two polyphagous insects. Sci Rep.

[45] Johnston PR, Rolff J (2015). Host and symbiont jointly control gut microbiota during complete metamorphosis. PLoS Pathog.

[46] Joosten L, van Veen JA (2011). Defensive properties of pyrrolizidine alkaloids against microorganisms. Phytochem Rev.

[47] Kaakoush NO (2015). Insights into the role of Erysipelotrichaceae in the human host. Front Cell Infect Microbiol.

[48] Kikuchi Y, Hosokawa T, Fukatsu T (2007). Insect-microbe mutualism without vertical transmission: a stinkbug acquires a beneficial gut symbiont from the environment every generation. Appl Environ Microbiol.

[49] Kembel SW, Cowan PD, Helmus MR, Cornwell WK, Morlon H, Ackerly DD, Blomberg SP, Webb CO (2010). Picante: R tools for integrating phylogenies and ecology. Bioinformatics.

[50] Klassen JL (2018). Defining microbiome function. Nat Microbiol.

[51] Kohl KD (2020). Ecological and evolutionary mechanisms underlying patterns of phylosymbiosis in host-associated microbial communities. Philos Trans R Soc B: Biol Sci.

[52] Koskella B, Bergelson J (2020). The study of host–microbiome (co)evolution across levels of selection. Philos Trans R Soc B: Biol Sci.

[53] Lanan MC, Rodrigues PAP, Agellon A, Jansma P, Wheeler DE (2016). A bacterial filter protects and structures the gut microbiome of an insect. ISME J.

[54] Lazzaro BP, Sackton TB, Clark AG (2006). Genetic variation in *Drosophila melanogaster* resistance to infection: a comparison across bacteria. Genetics.

[55] Li G, Zheng X, Zhu Y, Long Y, Xia X (2022). Bacillus symbiont drives alterations in intestinal microbiota and circulating metabolites of lepidopteran host. Environ Microbiol.

[56] Lindstedt C, Eager H, Ihalainen E, Kahilainen A, Stevens M, Mappes J (2011). Direction and strength of selection by predators for the color of the aposematic wood tiger moth. Behav Ecol.

[57] Lindstedt C, Suisto K, Burfield-Steel E, Winters AE, Mappes J (2020). Defense against predators incurs high reproductive costs for the aposematic moth *Arctia plantaginis*. Behav Ecol.

[58] Macke E, Tasiemski A, Massol F, Callens M, Decaestecker E (2017). Life history and eco-evolutionary dynamics in light of the gut microbiota. Oikos.

[59] Mallott EK, Amato KR (2021). Host specificity of the gut microbiome. Nat Rev Microbiol.

[60] Martin JD, Mundt JO (1972). Enterococci in insects. Appl Microbiol.

[61] Martínez-Solís M, Collado MC, Herrero S (2020). Influence of diet, sex, and viral infections on the gut microbiota composition of *Spodoptera exigua* caterpillars. Front Microbiol.

[62] Mason CJ, Ray S, Shikano I, Peiffer M, Jones AG, Luthe DS, Hoover K, Felton GW (2019). Plant defenses interact with insect enteric bacteria by initiating a leaky gut syndrome. Proc Natl Acad Sci.

[63] Mazumdar T, Teh BS, Murali A, Schmidt-Heck W, Schlenker Y, Vogel H, Boland W (2021). Transcriptomics reveal the survival strategies of *Enterococcus mundtii* in the gut of *Spodoptera littoralis*. J Chem Ecol.

[64] McMurdie PJ, Holmes S (2013). phyloseq: an R package for reproducible interactive analysis and graphics of microbiome census data. PLoS ONE.

[65] Mikaelyan A, Köhler T, Lampert N, Rohland J, Boga H, Meuser K, Brune A (2015). Classifying the bacterial gut microbiota of termites and cockroaches: a curated phylogenetic reference database (DictDb). Syst Appl Microbiol.

[66] Minard G, Tikhonov G, Ovaskainen O, Saastamoinen M (2019). The microbiome of the *Melitaea cinxia* butterfly shows marked variation but is only little explained by the traits of the butterfly or its host plant. Environ Microbiol.

[67] Moghadam NN, Thorshauge PM, Kristensen TN, de Jonge N, Bahrndorff S, Kjeldal H, Nielsen JL (2018). Strong responses of *Drosophila melanogaster* microbiota to developmental temperature. Fly.

[68] Näpflin K, Schmid-Hempel P (2016). Immune response and gut microbial community structure in bumblebees after microbiota transplants. Proc R Soc B: Biol Sci.

[69] Nokelainen O, Hegna RH, Reudler JH, Lindstedt C, Mappes J (2012). Trade-off between warning signal efficacy and mating success in the wood tiger moth. Proc R Soc B: Biol Sci.

[70] Nokelainen O, Lindstedt C, Mappes J (2013). Environment-mediated morph-linked immune and life-history responses in the aposematic wood tiger moth. J Anim Ecol.

[71] Nokelainen O, Galarza JA, Kirvesoja J, Suisto K, Mappes J (2022). Genetic colour variation visible for predators and conspecifics is concealed from humans in a polymorphic moth. J Evol Biol.

[72] Nyholm SV, Graf J (2012). Knowing your friends: invertebrate innate immunity fosters beneficial bacterial symbioses. Nat Rev Microbiol.

[73] Ohbayashi T, Takeshita K, Kitagawa W, Nikoh N, Koga R, Meng X-Y, Tago K, Hori T, Hayatsu M, Asano K, Kamagata Y, Lee BL, Fukatsu T, Kikuchi Y (2015). Insect’s intestinal organ for symbiont sorting. Proc Natl Acad Sci.

[74] Oksanen J, Blanchet FG, Friendly M, Kindt R, Legendre P, McGlinn D, Minchin PR, O'Hara RB, Simpson GL, Solymos P, Stevens MHH, Szoecs E, Wagner H. vegan: Community Ecology Package. R package version 2.5–7. https://CRAN.R-project.org/package=vegan (2020).

[75] Oliver KM, Moran NA, Hunter MS (2006). Costs and benefits of a superinfection of facultative symbionts in aphids. Proc R Soc B: Biol Sci.

[76] Osborn F, Berlioz L, Vitelli-Flores J, Monsalve W, Dorta B, Rodríguez Lemoine V (2002). Pathogenic effects of bacteria isolated from larvae of *Hylesia metabus* Crammer (Lepidoptera: Saturniidae). J Invertebrate Pathol.

[77] Paniagua Voirol LR, Frago E, Kaltenpoth M, Hilker M, Fatouros NE (2018). Bacterial symbionts in Lepidoptera: their diversity, transmission, and impact on the host. Front Microbiol.

[78] Parker ES, Moczek AP, Macagno ALM (2021). Reciprocal microbiome transplants differentially rescue fitness in two syntopic dung beetle sister species (Scarabaeidae: Onthophagus). Ecol Entomol.

[79] Parker BJ, McLean AHC, Hrček J, Gerardo NM, Godfray HCJ (2017). Establishment and maintenance of aphid endosymbionts after horizontal transfer is dependent on host genotype. Biol Let.

[80] Pietri JE, Tiffany C, Liang D (2018). Disruption of the microbiota affects physiological and evolutionary aspects of insecticide resistance in the German cockroach, an important urban pest. PLoS ONE.

[81] Pilon FM, Visôtto LE, Guedes RNC, Oliveira MGA (2013). Proteolytic activity of gut bacteria isolated from the velvet bean caterpillar *Anticarsia gemmatalis*. J Comp Physiol B.

[82] Pruesse E, Peplies J, Glöckner FO (2012). SINA: accurate high-throughput multiple sequence alignment of ribosomal RNA genes. Bioinformatics.

[83] Quast C, Pruesse E, Yilmaz P, Gerken J, Schweer T, Yarza P, Peplies J, Glöckner FO (2013). The SILVA ribosomal RNA gene database project: improved data processing and web-based tools. Nucleic Acids Res.

[84] R Core Team. 2021. R: A language and environment for statistical computing. R Foundation for Statistical Computing, Vienna, Austria. https://www.R-project.org/.

[85] Risely A (2020). Applying the core microbiome to understand host–microbe systems. J Anim Ecol.

[86] Rojas B, Burfield-Steel E, Pakkanen H, Suisto K, Maczka M, Schulz S, Mappes J (2017). How to fight multiple enemies: target-specific chemical defences in an aposematic moth. Proc R Soc B: Biol Sci.

[87] Rojas B, Mappes J, Burdfield-Steel E (2019). Multiple modalities in insect warning displays have additive effects against wild avian predators. Behav Ecol Sociobiol.

[88] Rojas B, Gordon S, Mappes J (2015). Frequency-dependent activity in the aposematic wood tiger moth, *Parasemia plantaginis*. Curr Zool.

[89] Rudman SM, Greenblum S, Hughes RC, Rajpurohit S, Kiratli O, Lowder DB, Lemmon SG, Petrov DA, Chaston JM, Schmidt P (2019). Microbiome composition shapes rapid genomic adaptation of *Drosophila melanogaster*. Proc Natl Acad Sci.

[90] Ruokolainen L, Ikonen S, Makkonen H, Hanski I (2016). Larval growth rate is associated with the composition of the gut microbiota in the Glanville fritillary butterfly. Oecologia.

[91] Schimming O, Challinor VL, Tobias NJ, Adihou H, Grün P, Pöschel L, Richter C, Schwalbe H, Bode HB (2015). Structure, biosynthesis, and occurrence of bacterial pyrrolizidine alkaloids. Angew Chem Int Ed.

[92] Shao Y, Chen B, Sun C, Ishida K, Hertweck C, Boland W (2017). Symbiont-derived antimicrobials contribute to the control of the Lepidopteran gut microbiota. Cell Chem Biol.

[93] Shao Y, Arias-Cordero E, Guo H, Bartram S, Boland W (2014). In vivo pyro-SIP assessing active gut microbiota of the cotton leafworm, *Spodoptera littoralis*. PLoS ONE.

[94] Shapira M (2016). Gut microbiotas and host evolution: scaling up symbiosis. Trends Ecol Evol.

[95] Sievers F, Wilm A, Dineen D, Gibson TJ, Karplus K, Li W, Lopez R, McWilliam H, Remmert M, Söding J, Thompson JD, Higgins DG (2011). Fast, scalable generation of high-quality protein multiple sequence alignments using Clustal Omega. Mol Syst Biol.

[96] Siva-Jothy MT, Moret Y, Rolff J, Simpson SJ (2005). Insect immunity: an evolutionary ecology perspective. Advances in Insect Physiology.

[97] Smee MR, Baltrus DA, Hendry TA (2017). Entomopathogenicity to two hemipteran insects is common but variable across epiphytic *Pseudomonas syringae* strains. Front Plant Sci.

[98] Smee MR, Raines SA, Ferrari J (2021). Genetic identity and genotype × genotype interactions between symbionts outweigh species level effects in an insect microbiome. ISME J.

[99] Spor A, Koren O, Ley R (2011). Unravelling the effects of the environment and host genotype on the gut microbiome. Nat Rev Microbiol.

[100] Stagaman K, Burns AR, Guillemin K, Bohannan BJ (2017). The role of adaptive immunity as an ecological filter on the gut microbiota in zebrafish. ISME J.

[101] Stamatakis A (2014). RAxML version 8: a tool for phylogenetic analysis and post-analysis of large phylogenies. Bioinformatics.

[102] Stanley D, Hughes RJ, Geier MS, Moore RJ (2016). Bacteria within the gastrointestinal tract microbiota correlated with improved growth and feed conversion: challenges presented for the identification of performance enhancing probiotic bacteria. Front Microbiol.

[103] Staudacher H, Kaltenpoth M, Breeuwer JAJ, Menken SBJ, Heckel DG, Groot AT (2016). Variability of bacterial communities in the moth *Heliothis virescens* indicates transient association with the host. PLoS ONE.

[104] Stegen J, Lin X, Konopka A, Fredrickson JK (2012). Stochastic and deterministic assembly processes in subsurface microbial communities. ISME J.

[105] Stegen JC, Lin X, Fredrickson JK, Chen X, Kennedy DW, Murray CJ, Rockhold ML, Konopka A (2013). Quantifying community assembly processes and identifying features that impose them. ISME J.

[106] Tang X, Freitak D, Vogel H, Ping L, Shao Y, Cordero EA, Andersen G, Westermann M, Heckel DG, Boland W (2012). Complexity and variability of gut commensal microbiota in polyphagous Lepidopteran larvae. PLoS ONE.

[107] Tamariz J, Burgueño-Tapia E, Vázquez MA, Delgado F, Knölker H-J (2018). Chapter One: Pyrrolizidine Alkaloids. The Alkaloids: Chemistry and Biology.

[108] Tegtmeier D, Riese C, Geissinger O, Radek R, Brune A (2016). *Breznakia blatticola* gen. nov. sp. nov. and *Breznakia pachnodae* sp. nov., two fermenting bacteria isolated from insect guts, and emended description of the family Erysipelotrichaceae. Syst Appl Microbiol.

[109] Teh B-S, Apel J, Shao Y, Boland W (2016). Colonization of the intestinal tract of the polyphagous pest *Spodoptera littoralis* with the GFP-tagged indigenous gut bacterium *Enterococcus mundtii*. Front Microbiol.

[110] Thakur A, Dhammi P, Saini HS, Kaur S (2015). Pathogenicity of bacteria isolated from gut of *Spodoptera litura* (Lepidoptera: Noctuidae) and fitness costs of insect associated with consumption of bacteria. J Invertebr Pathol.

[111] Turnbaugh PJ, Bäckhed F, Fulton L, Gordon JI (2008). Diet-induced obesity is linked to marked but reversible alterations in the mouse distal gut microbiome. Cell Host Microbe.

[112] Untergasser A, Cutcutache I, Koressaar T, Ye J, Faircloth BC, Remm M, Rozen SG (2012). Primer3-new capabilities and interfaces. Nucleic Acids Res.

[113] van Opstal EJ, Bordenstein SR (2019). Phylosymbiosis impacts adaptive traits in *Nasonia* wasps. MBio.

[114] Vernier CL, Chin IM, Adu-Oppong B, Krupp JJ, Levine J, Dantas G, Ben-Shahar Y (2020). The gut microbiome defines social group membership in honey bee colonies. Sci Adv.

[115] Vilanova C, Baixeras J, Latorre A, Porcar M (2016). The generalist inside the specialist: gut bacterial communities of two insect species feeding on toxic plants are dominated by *Enterococcus* sp. Front Microbiol.

[116] Visôtto LE, Oliveira MGA, Guedes RNC, Ribon AOB, Good-God PIV (2009). Contribution of gut bacteria to digestion and development of the velvetbean caterpillar, *Anticarsia gemmatalis*. J Insect Physiol.

[117] Visôtto LE, Oliveira MGA, Ribon AOB, Mares-Guia TR, Guedes RNC (2009). Characterization and identification of proteolytic bacteria from the gut of the velvetbean caterpillar (Lepidoptera: Noctuidae). Environ Entomol.

[118] Walter J, Maldonado-Gómez MX, Martínez I (2018). To engraft or not to engraft: an ecological framework for gut microbiome modulation with live microbes. Curr Opin Biotechnol.

[119] Walters AW, Hughes RC, Call TB, Walker CJ, Wilcox H, Petersen SC, Rudman SM, Newell PD, Douglas AE, Schmidt PS, Chaston JM (2020). The microbiota influences the *Drosophila melanogaster* life history strategy. Mol Ecol.

[120] Webb CO, Ackerly DD, McPeek MA, Donoghue MJ (2002). Phylogenies and community ecology. Annu Rev Ecol Syst.

[121] Weldon SR, Russell JA, Oliver KM (2020). More is not always better: coinfections with defensive symbionts generate highly variable outcomes. Appl Environ Microbiol.

[122] Wickham H (2016). ggplot2: elegant graphics for data analysis.

[123] Winters AE, Lommi J, Kirvesoja J, Nokelainen O, Mappes J (2021). Multimodal aposematic defenses through the predation sequence. Front Ecol Evol.

[124] Xia X, Lan B, Tao X, Lin J, You M (2020). Characterization of *Spodoptera litura* gut bacteria and their role in feeding and growth of the host. Front Microbiol.

[125] Zhang C, Zhang M, Wang S, Han R, Cao Y, Hua W, Mao Y, Zhang X, Pang X, Wei C, Zhao G, Chen Y, Zhao L (2010). Interactions between gut microbiota, host genetics and diet relevant to development of metabolic syndromes in mice. ISME J.

[126] Zhang F, Cui B, He X, Nie Y, Wu K, Fan D, Feng B, Chen D, Ren J, Deng M, Li N, Zheng P, Cao Q, Yang S, He X, Liu Y, Nie Y, Zhou Y, Fan D, Wu K, Nie Y, Ji G, Li P, Cui B, Zhang F, FMT-standardization Study Group (2018). Microbiota transplantation: concept, methodology and strategy for its modernization. Protein Cell.

